# Identification and prognosis of low office and ambulatory blood pressure in patients with heart failure

**DOI:** 10.1080/07853890.2025.2583558

**Published:** 2025-11-07

**Authors:** Zhuojin Li, Qinglan Tao, Yutong Wu, Sha Hua, Qingchuan Li, Zhiyan Wang, Yanjia Chen, Yan Li, Zeping Qiu, Wei Jin

**Affiliations:** a Department of Cardiovascular Medicine, Ruijin Hospital, Shanghai Jiao Tong University School of Medicine, Shanghai, China; b Heart Failure Center, Ruijin Hospital Lu Wan Branch, Shanghai Jiao Tong University School of Medicine, Shanghai, China; c Department of Cardiovascular Medicine, Shanghai Institute of Hypertension, Shanghai Key Laboratory of Hypertension, National Research Centre for Translational Medicine, State Key Laboratory of Medical Genomics, Ruijin Hospital, Shanghai Jiatong University School of Medicine, Shanghai, China

**Keywords:** Heart failure, ambulatory blood pressure monitoring, low blood pressure, hypotension

## Abstract

**Background:**

Low blood pressure (BP) limits the up-titration of guideline-directed medical therapies (GDMTs) and predicts poor outcomes in heart failure (HF). We assessed the value of ambulatory BP monitoring (ABPM) in detecting low BP and its impact on GDMTs optimization and prognosis in HF.

**Methods:**

In 491 HF patients initiating GDMTs from the Risk Evaluation and Management in Heart Failure (REM-HF) study since April 2018 to December 2022, ABPM was measured in addition to office BP. Participants were classified as sustained low systolic BP (SBP) (24-hour and office SBP < 120 mmHg), masked low SBP (24-hour SBP < 120 mmHg, office SBP ≥ 120 mmHg), and no low SBP. The primary outcome was a composite of all-cause mortality and HF rehospitalization. GDMTs target dose achievement was assessed at 3 months. Logistic regression and Cox regression models were used to assess GDMTs optimization and outcomes across SBP groups.

**Results:**

Sustained, masked, and no low SBP were observed in 25.3%, 30.8%, and 44.0% of patients, respectively. Both sustained (OR 2.36, 95%CI 1.25–4.47) and masked low SBP (OR 2.32, 95%CI 1.11–4.87) groups were associated with lower likelihood of achieving GDMTs target doses. Over a median 21-month follow-up, all-cause mortality and HF rehospitalization rates were higher in sustained (HR 2.45, 95% CI 1.56–3.86) and masked low SBP (HR 1.68, 95% CI 1.08–2.62) groups. No difference was found in the target dose achievement and outcomes between the two low SBP groups.

**Conclusion:**

Sustained and masked low SBP were common in HF and both associated with GDMTs intolerance and adverse outcomes.

## Graphical Abstract

**Figure:**
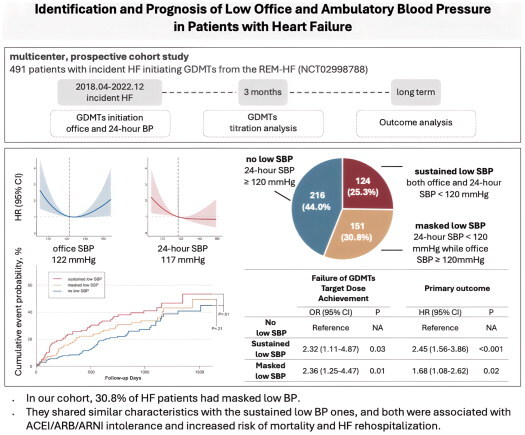

## Introduction

Evidence from landmark clinical trials has established ‘four pillars’ of pharmacological therapies for heart failure (HF), that includes an angiotensin receptor–neprilysin inhibitor (ARNI) or renin-angiotensin system (RAS) inhibitor, a beta-blocker, a mineralocorticoid receptor antagonist (MRA), and a sodium–glucose cotransporter 2 inhibitor (SGLT2i) [1,2]. HF guidelines in the U.S., Europe, and China all recommend early initiation of these agents and subsequent dose up-titration to reduce mortality and improve quality of life [3–5], which is why these drugs are collectively referred to as guideline-directed medical therapies (GDMTs). However, all GDMTs exert hypotensive effects [6]. This raises concerns about low blood pressure (BP) and relevant symptoms, leading to hesitancy in initiating or up-titrating these lifesaving medications [7,8]. Therefore, the identification of patients at risk of hypoperfusion may improve GDMTs implementation in clinical practice [9].

Ambulatory BP monitoring (ABPM) has established its clinical significance in hypertension management by providing detailed diurnal BP information, improving BP assessment accuracy, and better predicting adverse outcomes than office BP monitoring. ABPM can differentiate white-coat hypertension from office hypertension, and masked hypertension from office normotension. Masked hypertension is recognized as a discrete entity owing to its worse cardiovascular outcomes than true normotensives [10,11]. Although hypertension is one of the most common precursors of HF, elevation of systolic BP (SBP) is usually benign in patients with HF [12]. In contrast, lower SBP is associated with higher mortality and rehospitalization risks [13]. Low BP is commonly identified by office BP. Whether ABPM could help in detecting low SBP and improve risk classification beyond office BP among subjects with HF remains unknown.

Therefore, we established a multicenter, prospective Risk Evaluation and Management of Heart Failure (REM-HF) cohort [14,15], collecting ABPM data at GDMTs initiation to investigate: (i) the risk-related thresholds of low BP for office BP and ABPM; (ii) the prevalence of different low BP subgroups based on office BP and ABPM; and (iii) the association of different low SBP subgroups with GDMTs optimization and clinical outcomes.

## Methods

### Study design

REM-HF (NCT02998788) is a multicenter prospective cohort study designed to describe the clinical profiles, medication titration, and prognosis of HF. Enrolled HF patients visited at 15 and 30 days, 2, 3, 6, and 12 months, and every 6 months thereafter. GDMTs were optimized within 1–3 months by an HF specialist following the Chinese HF Guideline [5]. Dose adjustments were anticipated to be performed within the first three months (titration phase) after diagnosis and persisted in the subsequent period (stabilization phase) unless changes in clinical status were observed. Events were recorded at each clinic or during a telephonic visit.

The current study included adult subjects with REM-HF who had incident HF and initiated GDMTs at enrollment in the regional medical consortium of Huangpu District, Shanghai, China, between April 2018 and December 2022. Incident HF was defined as the first diagnosis of stage C symptomatic HF upon admission. Patients were considered to have new GDMTs initiation if they had never received any prescription of the following pharmacological class indicated by HF according to the electronic medical records: angiotensin-converting enzyme inhibitor (ACEI), angiotensin II receptor blocker (ARB), ARNI, beta-blocker, MRA, and SGLT2i. Key exclusion criteria were severe kidney disease with an estimated glomerular filtration rate (eGFR) of < 15 ml/min/1.73 m^2^, severe liver disease with Child-Pugh C, secondary hypertension, severe valvular disease, HF due to cardiac amyloidosis, and life expectancy of less than 1 year.

Eligible subjects conducted ABPM at baseline and those with invalid measurements were excluded. As ABPM is not a standard examination recommended by the HF guidelines, the results were not provided to physicians during routine follow-up visits to avoid the impact on drug initiation and titration. Baseline patient characteristics were collected from the electronic medical records. This study conformed to the principles outlined in the Declaration of Helsinki. This study was approved by the Institutional Review Board of Ruijin Hospital, Shanghai Jiao Tong University School of Medicine (2016-133). All the participants provided written informed consent.

### ABPM and office BP measurement

ABPM was performed using the Mobil-O-Graph (IEM, Germany) within 48 h after patients were diagnosed, independent of intravenous diuretic or inotropic, and restored daily physical activities. The monitors obtained BP readings every 20 min during the daytime (8 AM to 10 PM) and every 30 to 60 min during the nighttime (10 PM to 8 AM). ABPM was considered valid if there were at least 20 daytime readings and 7 nighttime readings. Office BP at baseline was measured in a seated position after a 10-minute rest using an automated sphygmomanometer (Omron HBP-1300), and the average value of three measurements taken at 1-minute intervals was recorded.

### GDMTs titration analysis

Only patients with valid medication and follow-up records within three months were included in this analysis to ensure that the maximum tolerated dose had been achieved. To describe changes in dose categories, we used four dose trajectory groups: (i) target dose achievement, (ii) initiation or dose increase, but target dose was not achieved, (iii) unchanged and suboptimal-target dose, and (iv) discontinuation or dose decrease. Medication dose changes that did not shift the dose category did not affect dose trajectory group assignment.

### Outcome

The primary outcome was a composite of all-cause mortality and hospitalization due to HF exacerbation. All outcome data were verified against the electronic medical records until August 2023. Only the first primary outcome was included in the analysis.

### Statistical analysis

Continuous variables were assessed for normality using the Shapiro-Wilk test and presented as mean ± standard deviation (SD) or median (interquartile range [IQR]), as appropriate. Between-group differences were compared using Student’s t-test or one-way analysis of variance (ANOVA) for normally distributed data, and Mann–Whitney U test or Kruskal-Wallis test for non-normally distributed data, with the Bonferroni method for post-hoc analysis. Categorical variables are shown as frequency (percentage) and were compared using the chi-square test. Univariate and multivariate Cox proportional hazards regression models were used to evaluate the association between SBP and outcomes. The proportional hazards assumption was evaluated using Schoenfeld residuals and revealed no violation of proportional hazards. Non-linear variables including N-terminal pro-brain natriuretic peptide (NT-proBNP), left ventricle ejection fraction (LVEF), and left ventricular end-diastolic dimension (LVEDD) were log-transformed to satisfy the linearity assumption in the Cox regression models. All potential predictors were assessed in univariate analysis, those with P values <0.05 were included in the multivariate analysis. Multicollinearity was evaluated using the Variance Inflation Factor (VIF), and variables with a VIF >10 were excluded. Model fit for Cox regression was assessed using pseudo R^2^. Bootstrap validation with 200 resamples was performed to obtain bias-corrected R^2^ for both univariate and multivariate models. Hazard Ratio (HR) for the primary outcome across the spectrum of SBP as a continuous variable was assessed using restricted cubic splines with three knots placed at the 10, 50, and 90th percentiles. Kaplan-Meier survival analysis with log-rank test was used to compare cumulative event rates between SBP groups. Subgroup analysis was performed to examine the homogeneity of the association between SBP and outcomes across subgroups, with interaction tests using likelihood ratio tests. Adjusted logistic regression models were used to examine the associations between SBP groups and 3-month GDMTs target dose achievement. A Sankey diagram was constructed using an online tool (https://app.rawgraphs.io) to describe patients’ dose category switching during titration. The data were analyzed using R version 4.2.3 (R Foundation for Statistical Computing, Vienna, Austria). A 2-sided P value <0.05 was considered statistical significance.

## Results

### Cohort characteristics according to office SBP

Of the 1041 incident HF patients with GDMTs initiation, 550 were excluded for reasons listed in Figure S1. The clinical characteristics of the remaining 491 patients are shown in Table S1, stratified by office SBP of 120 mmHg.

The mean (SD) age was 68.8 (16.8) years, and 280 (57.0%) were male. Overall, 170 (34.6%) patients had an office SBP < 120 mmHg, and they exhibited worse New York Heart Association (NYHA) functional class and higher NT-proBNP levels but similar echocardiographic parameters compared to those with office SBP ≥ 120 mmHg. There was no difference in the proportion of ACEI/ARB/ARNI initiators, whereas patients with an office SBP < 120 mmHg started at a lower dose.

### Correlation between outcomes and office and ambulatory SBP

Since there was no specific definition of low BP in HF, we analyzed the correlation between the primary outcome and SBP to determine the risk-related thresholds.

A total of 465 patients were followed with a median follow-up of 21 months, and 147 (31.6%) experienced the primary outcome of all-cause mortality (*n* = 67) or HF rehospitalization (*n* = 80). Multivariate Cox regression (adjusted for age, NYHA class, hemoglobin, eGFR, NT-proBNP, and the use of beta-blockers, MRA, diuretics, and nitrate) showed no significant association between office SBP and prognosis. However, the 24-hour SBP remained significantly associated with the primary outcome after adjustment, with an estimated 12% decrease in risk per 10 mmHg increase (HR 0.88, 95% CI 0.79–0.98, *p* = 0.02) (Tables S2 and S3).

**Figure 1.**
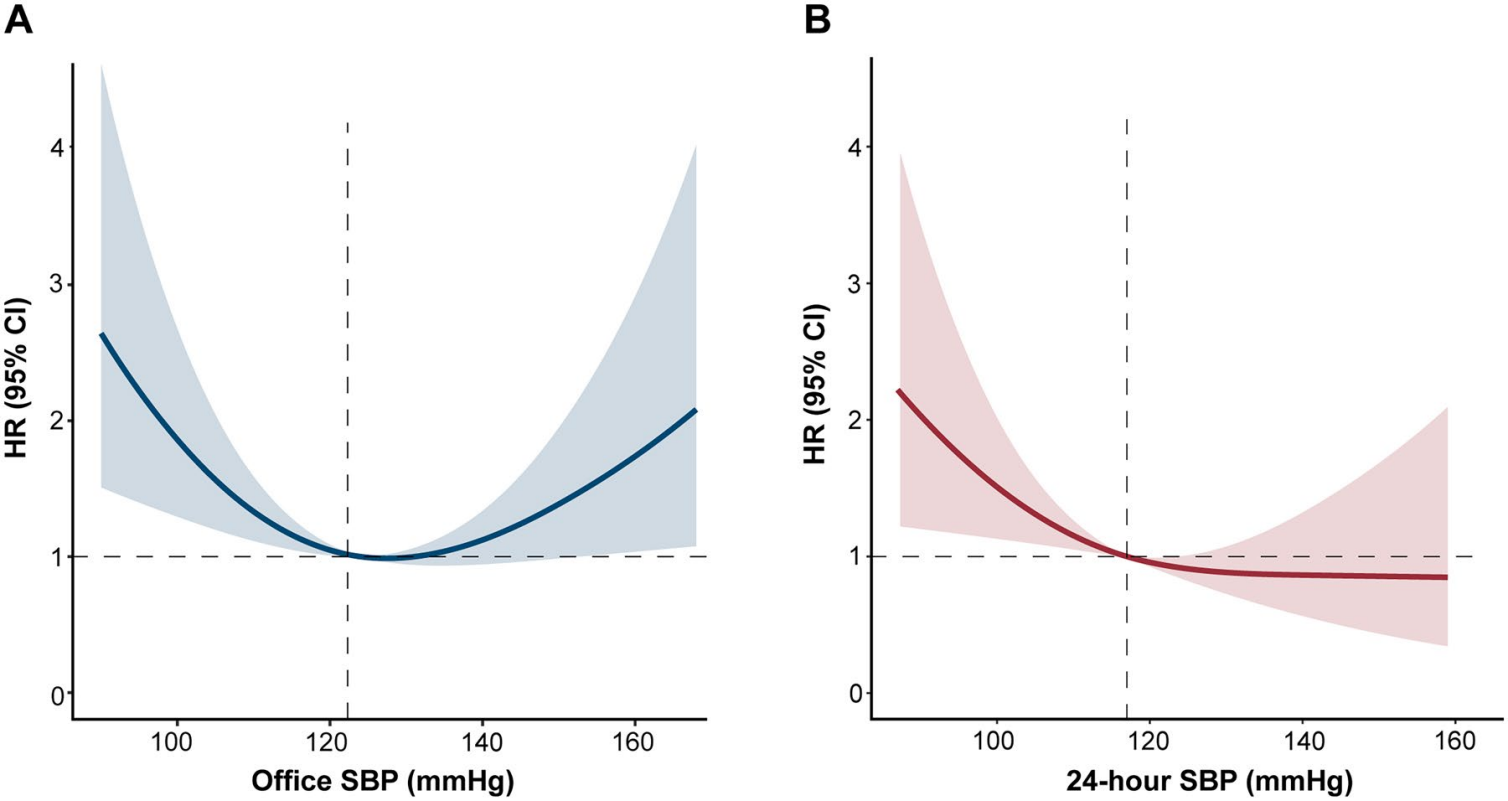
Association between office/24-hour SBP and the primary outcome. Restricted cubic spline curves depicting the nonlinear relationship (nonlinearity P <0.001) of office SBP (A) and linear relationship (nonlinearity P = 0.20) of 24-hour SBP (B) with the primary outcome of all-cause mortality and HF rehospitalization adjusted for age, NYHA class, hemoglobin, eGFR, NT-proBNP, and the use of beta-blockers, MRA, diuretics, and nitrates. HRs for the primary outcome were estimated using the spline-transformed SBP as a continuous variable. Solid lines represent HRs and shaded areas represent 95% CIs. CI: confidence interval; HR: hazard ratio; SBP: systolic blood pressure.

In the adjusted restricted cubic spline analysis, office SBP demonstrated a U-shaped relationship with the primary outcome (P for nonlinearity < 0.001). The lowest risk was found at 127 mmHg, and the risk increased significantly below 122 mmHg and above 153 mmHg (Figure 1A). In contrast, the curve for the 24-hour SBP and outcomes was linear (P for nonlinearity = 0.20), and the risk increased significantly below 117 mmHg (Figure 1B). Similar trends were observed for office and 24-hour diastolic BP (Figure S2).

These findings indicate a distinct prognostic value of ambulatory and office SBP in HF, but both were related to worse outcomes below approximately 120 mmHg. Therefore, we defined the risk-related threshold of low SBP in HF as SBP < 120 mmHg for both office and the 24-hour measurements.

### Masked and sustained low SBP in HF

Among 275 patients with low ambulatory SBP, 151 (54.9%) did not demonstrate low SBP based on office measurements. With office and 24-hour SBP measurements, patients were divided into three groups: sustained low SBP (both office and 24-hour SBP < 120 mmHg), masked low SBP (24-hour SBP < 120 mmHg and office SBP ≥ 120 mmHg), and no evidence of low SBP (24-hour SBP ≥ 120 mmHg). The masked low SBP group, where there was disagreement between office BP and ABPM, was of particular interest and suggested a population with unrecognized risk in routine practice.

A total of 124 (25.3%), 151 (30.8%), and 216 (44.0%) patients were grouped into sustained low SBP, masked low SBP, and no low SBP groups, respectively, in our cohort (Table 1). No between-group differences were observed in NT-proBNP levels. In general, the masked low SBP group demonstrated traits similar to those of individuals with a sustained low SBP. These two groups had comparable left ventricle ejection fraction (55.0% vs. 52.5%) and left ventricular end-diastolic dimension (LVEDD, 51.0 mm vs 54.0 mm), both worse than the no low SBP group. Despite the similar GDMTs initiation rates, patients with masked low SBP often started with higher ACEI/ARB/ARNI doses than those with sustained low SBP.

**Table 1.** Characteristics of the study population by SBP groups.

	Sustained low SBP (*n* = 124)	Masked low SBP (*n* = 151)	No low SBP (*n* = 216)	*P*	*P ^a^*	*P* ^b^	*P* ^c^
Age, years	64.7 ± 16.8	68.4 ± 16.9	71.4 *±* 16.3	0.001	0.001	0.26	0.20
Sex, male (%)	75 (60.5)	89 (58.9)	116 (53.7)	0.41	NA	NA	NA
NYHA Class, III/IV (%)	84 (67.7)	92 (60.9)	139 (64.4)	0.53	NA	NA	NA
Smoking (%)	21 (16.9)	19 (12.6)	31 (14.4)	0.59	NA	NA	NA
Comorbidities (%)							
Ischemic heart disease	43 (34.7)	65 (43.0)	108 (50.0)	0.02	0.03	0.68	0.59
Hypertension	52 (41.9)	95 (62.9)	178 (82.4)	<0.001	<0.001	<0.001	0.002
Diabetes	32 (25.8)	33 (21.9)	85 (39.4)	<0.001	0.05	0.002	1.00
Atrial fibrillation	54 (43.5)	47 (31.1)	78 (36.1)	0.10	NA	NA	NA
Laboratory measurements							
Hemoglobin, g/L	130.3 ± 25.1	129.3 ± 24.0	126.9 ± 21.1	0.40	0.63	1.00	1.00
eGFR, ml/min/1.73m^2^	75.6 ± 23.4	71.5 ± 22.0	72.5 ± 23.6	0.34	0.75	1.00	0.48
Serum sodium, mmol/L	140.8 ± 3.2	140.7 ± 3.2	140.8 ± 3.8	0.99	1.00	1.00	1.00
Serum potassium, mmol/L	4.0 ± 0.5	4.0 ± 0.4	3.9 ± 0.4	0.09	0.64	0.09	1.00
Fasting glucose, mmol/L	5.7 ± 2.0	6.1 ± 2.3	6.1 ± 2.9	0.45	0.71	1.00	0.89
Total cholesterol, mmol/L	4.1 ± 1.1	4.0 ± 1.3	4.4 ± 1.4	0.03	0.12	0.06	1.00
NT-proBNP, pg/mL	1525.0 (546.6–3408.8)	1223.0 (582.5–2768.8)	1156.5 (509.6–2402.5)	0.44	NA	NA	NA
TTE parameters							
LVEDD, mm	54.0 (47.0–66.0)	51.0 (47.0–63.0)	50.0 (45.0–57.5)	0.002	0.004	0.03	1.00
LVEF, %	52.5 (36.0–63.0)	55.0 (38.5–64.5)	62.0 (46.5–66.0)	<0.001	<0.001	0.01	0.83
BP parameters, mmHg							
Office SBP	104.4 ± 9.8	132.6 ± 11.5	132.7 ± 20.3	<0.001	<0.001	1.00	<0.001
Office DBP	63.9 ± 10.5	74.1 ± 11.2	73.3 ± 14.7	<0.001	<0.001	1.00	<0.001
24-hour SBP	101.8 ± 9.3	108.5 ± 8.5	134.5 ± 12.7	<0.001	<0.001	<0.001	<0.001
24-hour DBP	60.5 ± 7.6	62.9 ± 9.6	72.0 ± 11.3	<0.001	<0.001	<0.001	0.13
Daytime SBP	102.4 ± 9.5	108.4 ± 11.4	134.7 ± 13.0	<0.001	<0.001	<0.001	<0.001
Daytime DBP	61.0 ± 8.0	64.0 ± 8.8	72.3 ± 11.5	<0.001	<0.001	<0.001	0.04
Nighttime SBP	100.2 ± 11.7	107.2 ± 11.2	135.1 ± 17.0	<0.001	<0.001	<0.001	<0.001
Nighttime DBP	59.3 ± 8.9	62.1 ± 8.9	71.6 ± 13.0	<0.001	<0.001	<0.001	0.13
Medication at discharge (%)							
ACEI/ARB/ARNI	107 (86.3)	129 (85.4)	191 (88.4)	0.68	NA	NA	NA
≥50% ACEI/ARB/ARNI target dose	35 (28.2)	64 (42.4)	131 (60.6)	<0.001	<0.001	0.003	0.06
Beta-blockers	110 (88.7)	125 (82.8)	170 (78.7)	0.07	NA	NA	NA
MRA	87 (70.2)	106 (70.2)	149 (69.0)	0.96	NA	NA	NA
SGLT2i	27 (21.8)	25 (16.6)	36 (16.7)	0.43	NA	NA	NA
Diuretic	69 (55.6)	96 (63.6)	143 (66.2)	0.15	NA	NA	NA
Calcium channel blockers	8 (6.5)	26 (17.2)	87 (40.3)	<0.001	<0.001	<0.001	0.04
Nitrate	15 (12.1)	24 (15.9)	63 (29.2)	<0.001	0.002	0.01	1.00

Values are expressed as mean ± SD, median (IQR), or n (%). Between-group differences were estimated using one-way analysis of variance (ANOVA), Kruskal-Wallis test, or chi-square test with the Bonferroni method for post-hoc analysis. Statistical significance was set at *p* < 0.05. **^a^**Sustained low SBP vs. no low SBP. ^b^ Masked low SBP vs. no low SBP. ^c^ Sustained low SBP vs. masked low SBP.

ACEI: angiotensin-converting enzyme inhibitor; ARB: angiotensin II receptor blocker; ARNI: angiotensin receptor–neprilysin inhibitors; DBP: diastolic blood pressure; eGFR: estimated glomerular filtration rate; LVEDD: left ventricular end-diastolic dimension; LVEF: left ventricular ejection fraction; MRA: mineralocorticoid receptor antagonist; NT-proBNP: N-terminal pro-brain natriuretic peptide; NYHA: New York Heart Association; SBP: systolic blood pressure; SGLT2i: sodium-glucose cotransporter-2 inhibitors; TTE: transthoracic echocardiography.

Our study revealed that nearly one-third of new-onset HF patients presented with what we called ‘masked low BP.’ They shared similar clinical features with ‘sustained low BP’ patients but were always treated as normotensive patients in practice.

### Effect of masked and sustained low SBP on GDMTs titration

To analyze the impact of masked and sustained low SBP on actual GDMTs prescription, 15 patients who died within 3 months and 124 patients without qualified medication records to ensure credible titration were excluded. The characteristics of the SBP groups of the remaining 352 patients are presented in Table S4.

At the end of titration, the median (IQR) dose for ACEI/ARB/ARNI, shown as a percentage of the guideline recommended dose, was 50.0 (25.0–75.0) % in the masked low SBP group, lower than the 50.0 (50.0–100.0) % in the sustained low SBP group (*p* < 0.001), but similar to the 31.3 (25.0–75.0%) in the no low SBP group (*p* = 1.00).

**Figure 2.**
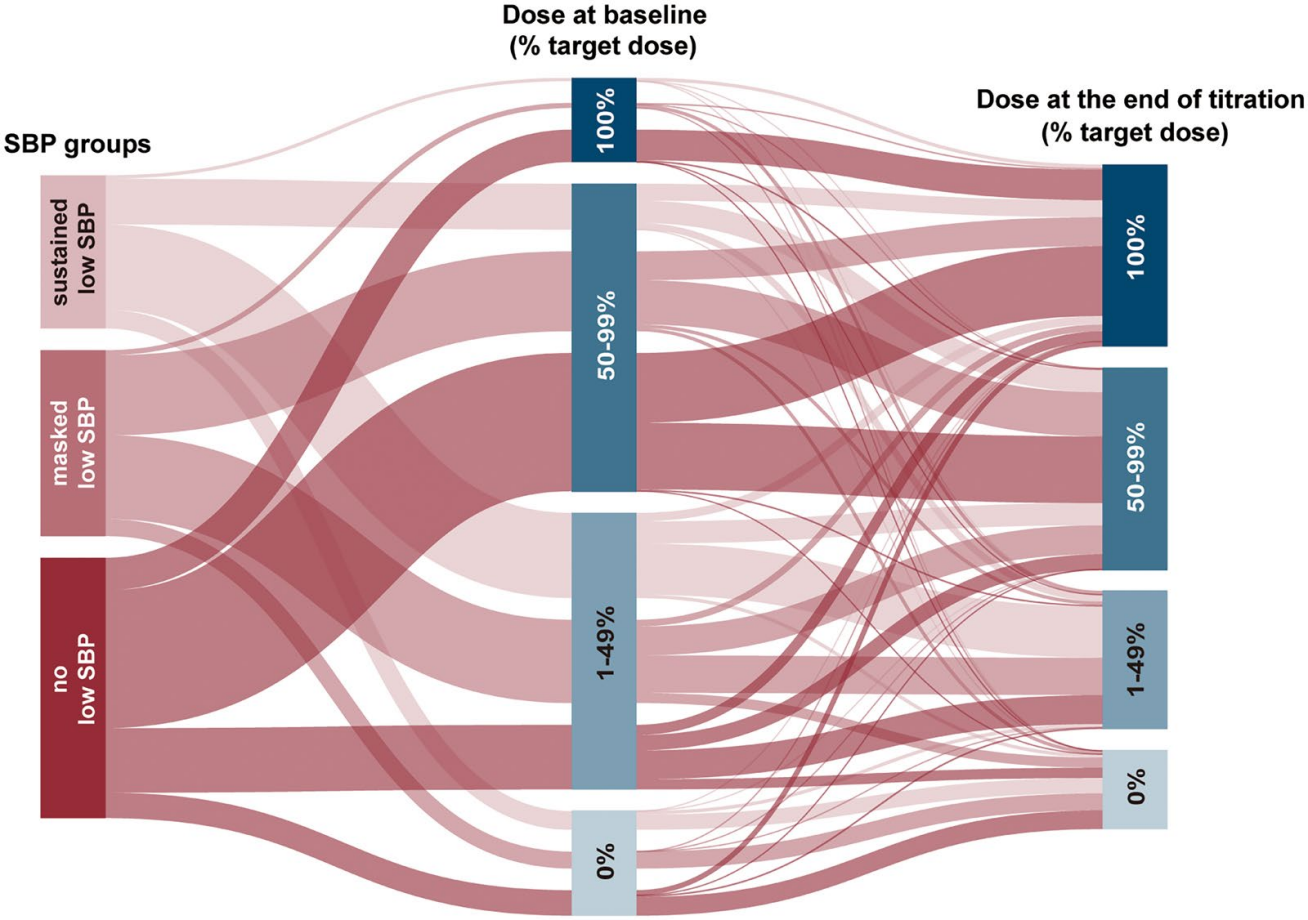
GDMTs dose trajectory by SBP groups over 3-month titration. The ACEI/ARB/ARNI dose switching over a 3-month titration of patients with sustained low SBP, masked low SBP, or without low SBP is displayed by the Sankey diagram. The ACEI/ARB/ARNI dose is shown as a percentage of the target dose. SBP: systolic blood pressure.

The switch between dose categories before and after titration is displayed in the Sankey diagram (Figure 2). As shown in Table S5, 32 (35.6%), 39 (35.8%), and 78 (51.0%) patients in the sustained, masked, and no low SBP groups, respectively, newly initiated or up-titrated ACEI/ARB/ARNI (*p* = 0.02). GDMT intolerance (medication discontinuation or dose reduction) was higher in the masked low SBP group (11.0%) than in the sustained (6.7%) or no (5.2%) low SBP groups. Figure S3 shows individual office SBP changes after GDMTs titration; more patients with masked low SBP experienced a decrease than patients with sustained low SBP (62.7% vs. 29.0%). In fact, the majority of patients with sustained low SBP showed an SBP increase after treatment. Both the sustained (OR 2.32, 95% CI 1.11–4.87, *p* = 0.03) and masked (OR 2.36, 95% CI 1.25–4.47, *p* = 0.01) low SBP groups were independently associated with failure of ACEI/ARB/ARNI dose achievement after adjustment, with no significant difference between them (*p* = 0.97) (Table 2 and Table S6).

**Table 2.** The association of the failure of ACEI/ARB/ARNI target dose achievement and the primary outcome with SBP groups.

	Failure of ACEI/ARB/ARNI target dose achievement				Primary outcome			
	Unadjusted		Adjusted ^a^		Unadjusted		Adjusted **^b^**	
	OR (95% CI)	*P*	OR (95% CI)	*P*	HR (95% CI)	*P*	HR (95% CI)	*P*
No low SBP	Reference	NA	Reference	NA	Reference	NA	Reference	NA
Sustained low SBP	3.44 (1.85–6.37)	<0.001	2.32 (1.11–4.87)	0.03	1.68 (1.13–2.51)	0.01	2.45 (1.56–3.86)	<0.001
Masked low SBP	3.16 (1.80–5.57)	<0.001	2.36 (1.25–4.47)	0.01	1.30 (0.87–1.94)	0.21	1.68 (1.08–2.62)	0.02

Logistic regression for the failure of ACEI/ARB/ARNI target dose achievement and Cox Regression for the Primary Outcome with SBP groups. Statistical significance was set at *p* < 0.05. ^a^ adjusted variables included hypertension, LVEF, and CCB use. **^b^** adjustment variables included age, NYHA class, hemoglobin, eGFR, NT-proBNP, and the use of beta-blockers, MRA, diuretics, and nitrate.

CI: conference interval; HR: hazard ratio; OR: odds ratio; SBP: systolic blood pressure.

Taken together, HF patients with sustained or masked low SBP faced a greater risk of GDMTs intolerance and often achieved suboptimal doses than those genuinely without low SBP. Given the similar office SBP levels between the masked and no low SBP groups at first look (129 mmHg vs. 130 mmHg, *p* = 0.10), masked low BP warrants more attention from HF specialists.

### Effect of masked and sustained low SBP on clinical outcomes

**Figure 3.**
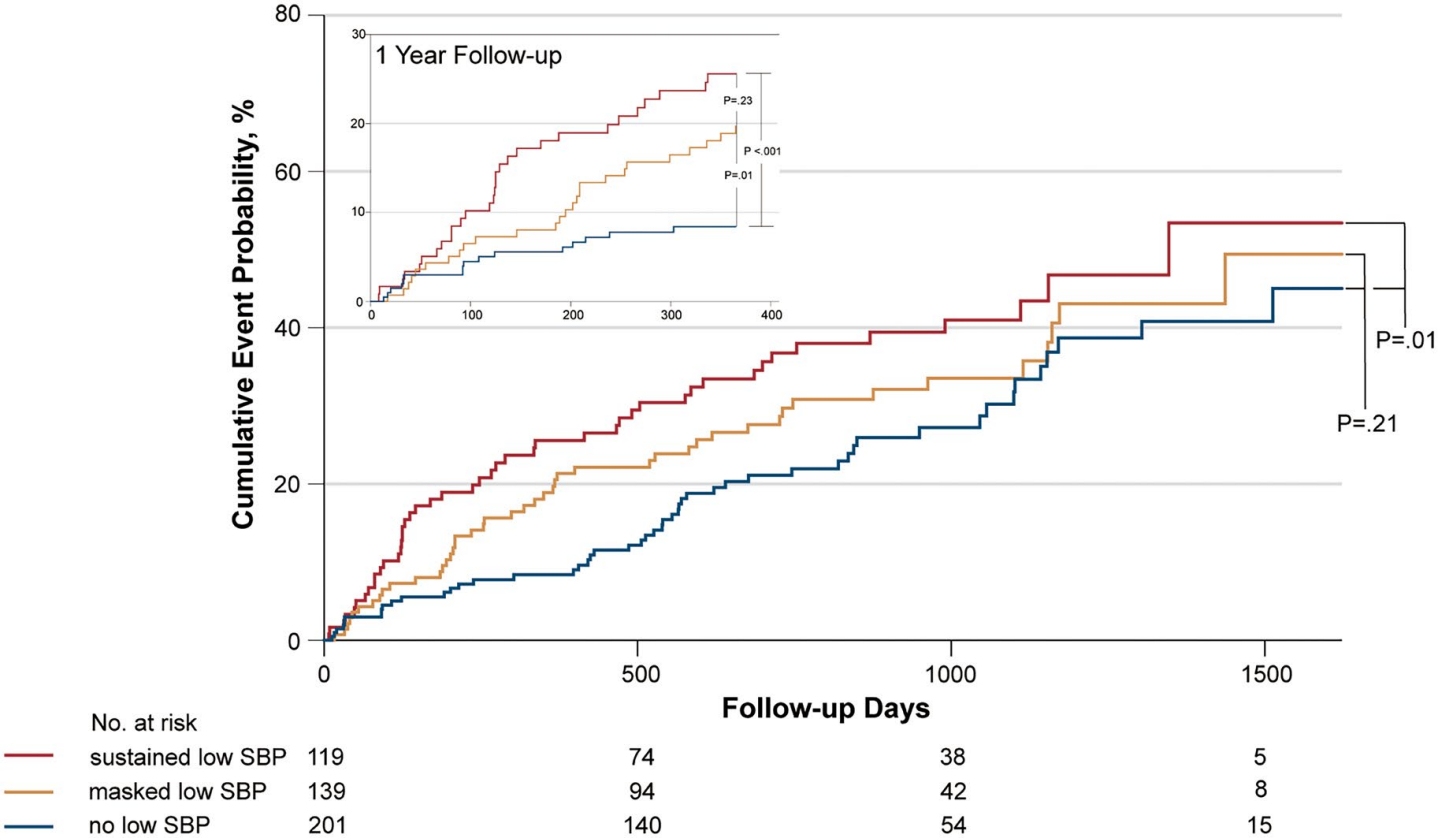
One-year or long-term primary composite outcome by SBP groups. Kaplan–Meier curves depicting cumulative incidence of the one-year or long-term primary composite outcome (first occurrence of all-cause mortality and HF rehospitalization). The P value representing the difference between the SBP groups was estimated using the log-rank test.

After 1-year follow-up, the cumulative incidences of primary outcome were 24.4%, 18.7%, and 8.0% in sustained, masked, and no low SBP group respectively. The specific events for the outcomes were listed in Table S7. Over 60 months of follow-up, more adverse events occurred in patients with sustained or masked low SBP than those without low SBP (Figure 3). Fully adjusted Cox regression showed a 2.5-fold increased risk in sustained low SBP group (HR 2.45, 95% CI 1.56–3.86, *p* < 0.001) and a 1.7-fold increase in masked low SBP group (HR 1.68, 95% CI 1.08–2.62, *p* = 0.02) versus the no low SBP group, with no survival difference between the two low SBP groups (Table 2). Subgroup analyses showed no heterogeneity in the association between SBP groups and outcomes (Figure S4). In our cohort, 118 (24.0%) patients had HFrEF, and 314 (64.0%) had HFpEF. Both sustained and masked low SBP were linked to poor medication titration and worse outcomes across the two populations, although the association of masked low SBP being less pronounced in HFpEF (Table S8). Sensitivity analyses using a different threshold for low office SBP (less than 130 mmHg) also confirmed these findings (Table S9).

## Discussion

This multicenter, prospective study highlights the additional value of ABPM in detecting clinically relevant HF in patients with low BP. Using a risk-related threshold, we observed that about 30% of HF patients had low ambulatory BP undetectable by routine clinic BP measurement, as so called ‘masked low BP.’ This masked low BP group shared similar clinical characteristics with the sustained low SBP group and faced a higher risk of 1-year, and overall all-cause mortality and HF rehospitalization. Office BP showed a U-shaped relationship with outcomes, whereas 24-hour SBP demonstrated a linear association, suggesting that ABPM may more accurately reflect low BP status in HF patients. These findings emphasize the need to recognize ‘masked low BP’ as a discrete entity, more like the ‘genuinely low BP,’ yet overlooked by a conventional clinical BP measurement.

Low BP has an adverse prognostic impact in patients with HF, irrespective of LVEF. According to the OPTIMIZE-HF registry, an SBP <130 mmHg in HF with reduced ejection fraction (HFrEF) and <120 mmHg in HF with preserved ejection fraction (HFpEF) was associated with increased risks of death and HF hospitalization [16,17]. Nevertheless, it should be acknowledged that HFrEF and HFpEF differ in their pathophysiology and therapeutic strategies. For patients with HFrEF, current guidelines suggest that GDMTs should be optimized even in the presence of slight hypotension [3,4]. However, physicians are often reluctant to prescribe these life-saving agents in the context of low BP, due to the potential risk of additional BP lowering. This hesitancy may hinder GDMTs application in clinical practice and limits patients from achieving maximal therapeutic benefit. With respect to HFpEF, SGLT2 inhibitor is the only treatment shown to improve mortality and morbidity, despite the use of ARNI in those with an LVEF ‘less than normal’ has been endorsed by the administration [18]. Therefore, the clinical significance of GDMTs titration is less certain in HFpEF, and low BP itself may represent an independent risk factor in this population [19]. This indicates the distinct prognostic value of low BP in HFpEF from that in HFrEF and further supports the utility of ABPM in identifying high-risk patients.

ABPM is an established tool for comprehensive and reproducible BP evaluation through repetitive measurements and the inclusion of circadian rhythm [20,21]. Masked hypertension, defined as normal in-office BP but elevated out-of-office BP, is associated with early organ damage and has gained attention for its timely identification [22,23]. Although lowering BP clearly improved prognosis in the general population [24], HF management prioritizes reducing mortality with trial-proven therapies [25]. The hypotensive effect thus became an unwanted bonus for GDMTs in HF [26]. The STRONG-HF study demonstrated that rapid up-titration of GDMTs (including ACEI, ARB, ARNI, beta-blocker, and MRA) to target doses lowered the risks of all-cause mortality and rehospitalization in patients with acute HF, with consistent benefits observed in both the LVEF ≤40% and LVEF >40% subgroups [27]. These findings cannot be extrapolated to all patients, as it excluded patients with SBP <100 mmHg or intolerance to the 50% recommended dose. In addition, patients in the real world are older, have more comorbidities, and poorer general health, raising the concern that further drug-induced BP lowering might be detrimental. Therefore, we focused on the detection of low BP by ABPM in HF, which may provide a glimpse into who will suffer organ hypoperfusion before the consequences occur.

We unexpectedly discovered an inconsistency between the office and ambulatory SBP, which we termed ‘masked low BP.’ Divisón et al. previously described a similar condition in treated hypertensive patients with older age or coronary heart disease, as office BP ≥110/70 or 120/70 mmHg but 24-hour BP <100/60 or <110/60 mmHg [28]. These thresholds, derived from the PROVE IT-TIMI and CLARIFY trials, which showed SBP <110 or 120 mmHg related to increased cardiovascular events in patients with coronary artery disease, cannot be directly applied to HF [29,30]. In our study, both patients with sustained and masked low SBP showed intolerance to ACEI/ARB/ARNI and a higher risk of the primary outcome. Interestingly, most of the sustained low SBP group experienced an increase in office BP during GDMTs, indicating that even those with a lower baseline BP can draw treatment benefits from GDMTs [31,32] since SBP is primarily dependent on left ventricular stroke volume in HF. Meanwhile, the masked low SBP group demonstrated a decrease in office BP after treatment, which still needs to be understood and might be partially explained by the ‘regression to the mean’ principle.

A potential mechanism for masked low BP may be the unique form of the ‘white coat effect.’ Typically, the white coat effect refers to elevated BP inside the clinic but normal BP outside due to stressful stimuli in office settings by sympathetic activation [33]. Sympathetic nervous system activation is a pathophysiological hallmark of HF and is characterized by elevated cardiac norepinephrine spillover and increased muscle sympathetic nerve activity (MSNA) [34]. However, sustained activation during disease progression can cause downregulation in density and desensitization of the β-adrenergic receptors on the cardiac surface, known as cardiac sympathetic denervation [35]. Increased MSNA levels may also reduce α-adrenoceptor–mediated vasoconstriction. A recent clinical study showed that sympathetic-BP transduction was lower in patients with HF, particularly those with elevated MSNA [36]. Taken together, we hypothesize that masked low SBP is an early stage of sustained low SBP, where true low BP is masked by transient BP elevation during office visits, with preserved sympathetic BP transduction. As HF advances, this mechanism diminishes, leading to a more pronounced decrease in SBP. In addition to the elevated office BP explained by white coat effect, several mechanisms may account for low BP detected by ABPM. One possibility is orthostatic hypotension, which is frequently observed in patients with HF and may contribute to lower BP readings during daily activities [37]. Another possible explanation is that reduced nocturnal BP on ABPM results from exaggerated nighttime dipping, which may also serve as a marker of impaired sympathetic activation [38]. Both mechanisms highlight the importance of ABPM in capturing dynamic BP fluctuations that cannot be identified through office measurements alone.

Some limitations must be considered when interpreting the results of this study. As an observational cohort, it cannot fully eliminate selection bias or provide guidance for GDMTs adjustment strategies. The threshold for low BP was determined based on a risk-related method without considering symptoms such as dizziness or fatigue. Whether an optimal BP target should be guided by a predefined threshold or by clinical judgement based on symptoms requires further investigation. Although our analyses suggested similar associations between SBP groups with medication optimization and clinical outcomes in both HFrEF and HFpEF, the relatively small proportion of HFrEF patients (24%) limited the statistical power to draw definitive conclusions for this subgroup. Other limitations include the generalizability to other countries or ethnicities with different diets and lifestyles. A properly designed prospective randomized clinical trial is needed to confirm the performance of ABPM and masked low BP in patients with HF.

## Conclusions

With a comprehensive office and ambulatory BP assessment, we observed that 30.8% of the patients with HF had masked low BP. These patients shared similar characteristics with sustained low BP patients, and both groups were associated with ACEI/ARB/ARNI intolerance and increased risk of mortality and HF rehospitalization. Our findings imply that ‘masked low BP’ should be treated as an important clinical entity in the management of HF and further research in these patients is warranted.

## Supplementary Material

Supplemental Material

## Data Availability

The data that support the findings of this study are available from the corresponding author upon reasonable request.

## References

[1] CONSENSUS Trial Study Group. Effects of enalapril on mortality in severe congestive heart failure. Results of the Cooperative North Scandinavian Enalapril Survival Study (CONSENSUS). N Engl J Med. 1987;316(23):1429–1435. 2883575 10.1056/NEJM198706043162301

[2] McMurray JJV, Packer M, Desai AS, et al. Dual angiotensin receptor and neprilysin inhibition as an alternative to angiotensin-converting enzyme inhibition in patients with chronic systolic heart failure: rationale for and design of the Prospective comparison of ARNI with ACEI to Determine Impact on Global Mortality and morbidity in Heart Failure trial (PARADIGM-HF). Eur J Heart Fail. 2013;15(9):1062–1073. doi: 10.1093/eurjhf/hft052. 23563576 PMC3746839

[3] McDonagh TA, Metra M, Adamo M, et al. 2021 ESC Guidelines for the diagnosis and treatment of acute and chronic heart failure. Eur Heart J. 2021;42(36):3599–3726. doi: 10.1093/eurheartj/ehab368. 34447992

[4] Heidenreich PA, Bozkurt B, Aguilar D, et al. 2022 AHA/ACC/HFSA guideline for the management of heart failure: a report of the American College of Cardiology/American Heart Association Joint Committee on Clinical Practice Guidelines. Circulation. 2022;145(18):e895–e1032. 35363499 10.1161/CIR.0000000000001063

[5] Zhonghua X, Xue G, Bing ZZ. [Chinese guidelines for the diagnosis and treatment of heart failure 2018]. 2018;46(10):760–789. 10.3760/cma.j.issn.0253-3758.2018.10.004 30369168

[6] Cautela J, Tartiere J-M, Cohen-Solal A, et al. Management of low blood pressure in ambulatory heart failure with reduced ejection fraction patients. Eur J Heart Fail. 2020;22(8):1357–1365. doi: 10.1002/ejhf.1835. 32353213 PMC7540603

[7] Savarese G, Bodegard J, Norhammar A, et al. Heart failure drug titration, discontinuation, mortality and heart failure hospitalization risk: a multinational observational study (US, UK and Sweden). Eur J Heart Fail. 2021;23(9):1499–1511. doi: 10.1002/ejhf.2271. 34132001

[8] Savarese G, Kishi T, Vardeny O, et al. Heart failure drug treatment-inertia, titration, and discontinuation: a multinational observational study (EVOLUTION HF). JACC Heart Fail. 2023;11(1):1–14. doi: 10.1016/j.jchf.2022.08.009. 36202739

[9] Komajda M, Schöpe J, Wagenpfeil S, et al. Physicians’ guideline adherence is associated with long-term heart failure mortality in outpatients with heart failure with reduced ejection fraction: the QUALIFY international registry. Eur J Heart Fail. 2019;21(7):921–929. doi: 10.1002/ejhf.1459. 30933403

[10] Reboussin DM, Allen NB, Griswold ME, et al. Systematic review for the 2017 ACC/AHA/AAPA/ABC/ACPM/AGS/APhA/ASH/ASPC/NMA/PCNA Guideline for the prevention, detection, evaluation, and management of high blood pressure in adults: a report of the American College of Cardiology/American Heart Association task force on clinical practice guidelines. Circulation. 2018;138(17):e595–e616. doi: 10.1161/CIR.0000000000000601. 30354656

[11] Zhang D-Y, Cheng Y-B, Guo Q-H, et al. Treatment of masked hypertension with a chinese herbal formula: a randomized, placebo-controlled trial. Circulation. 2020;142(19):1821–1830. doi: 10.1161/CIRCULATIONAHA.120.046685. 33019798

[12] Lee MH, Leda M, Buchan T, et al. Prognostic value of blood pressure in ambulatory heart failure: a meta-analysis and systematic review. Ambulatory blood pressure predicts heart failure prognosis. Heart Fail Rev. 2022;27(2):455–464. doi: 10.1007/s10741-021-10086-w. 33682033

[13] Ventura HO, Messerli FH, Lavie CJ. Observations on the blood pressure paradox in heart failure. Eur J Heart Fail. 2017;19(7):843–845. doi: 10.1002/ejhf.818. 28370872

[14] Hua S, Lv B, Qiu Z, et al. Microbial metabolites in chronic heart failure and its common comorbidities. EMBO Mol Med. 2023;15(6):e16928. doi: 10.15252/emmm.202216928. 37155563 PMC10245034

[15] Chen Y, Qiu Z, Jiang J, et al. Outcomes of spironolactone withdrawal in dilated cardiomyopathy with improved ejection fraction. Front Cardiovasc Med. 2021;8:725399. doi: 10.3389/fcvm.2021.725399. 34604354 PMC8481596

[16] Arundel C, Lam PH, Gill GS, et al. Systolic blood pressure and outcomes in patients with heart failure with reduced ejection fraction. J Am Coll Cardiol. 2019;73(24):3054–3063. doi: 10.1016/j.jacc.2019.04.022. 31221253 PMC10656059

[17] Tsimploulis A, Lam PH, Arundel C, et al. Systolic blood pressure and outcomes in patients with heart failure with preserved ejection fraction. JAMA Cardiol. 2018;3(4):288–297. doi: 10.1001/jamacardio.2017.5365. 29450487 PMC5875342

[18] Lund LH, Crespo-Leiro MG, Laroche C, et al. Heart failure in Europe: guideline-directed medical therapy use and decision making in chronic and acute, pre-existing and de novo, heart failure with reduced, mildly reduced, and preserved ejection fraction - the ESC EORP Heart Failure III Registry. Eur J Heart Fail. 2024;26(12):2487–2501. doi: 10.1002/ejhf.3445. 39257278 PMC11683873

[19] Foà A, Vaduganathan M, Claggett BL, et al. Sacubitril/valsartan-related hypotension in patients with heart failure and preserved or mildly reduced ejection fraction. J Am Coll Cardiol. 2024;83(18):1731–1739. doi: 10.1016/j.jacc.2024.02.035. 38537919

[20] Huang Q-F, Yang W-Y, Asayama K, et al. Ambulatory blood pressure monitoring to diagnose and manage hypertension. Hypertension. 2021;77(2):254–264. doi: 10.1161/HYPERTENSIONAHA.120.14591. 33390042 PMC7803442

[21] Schutte AE, Kollias A, Stergiou GS. Blood pressure and its variability: classic and novel measurement techniques. Nat Rev Cardiol. 2022;19(10):643–654. doi: 10.1038/s41569-022-00690-0. 35440738 PMC9017082

[22] Franklin SS, O’Brien E, Staessen JA. Masked hypertension: understanding its complexity. Eur Heart J. 2017;38(15):1112–1118. doi: 10.1093/eurheartj/ehw502. 27836914

[23] Fu X, Ren H, Xie J, et al. Association of nighttime masked uncontrolled hypertension with left ventricular hypertrophy and kidney function among patients with chronic kidney disease not receiving dialysis. JAMA Netw Open. 2022;5(5):e2214460. doi: 10.1001/jamanetworkopen.2022.14460. 35616936 PMC9136624

[24] Lewis CE, Fine LJ, Beddhu S, et al. Final report of a trial of intensive versus standard blood-pressure control. N Engl J Med. 2021;384(20):1921–1930. 34010531 10.1056/NEJMoa1901281 PMC9907774

[25] Fu M, Swedberg K. Enhancing implementation of evidence-based heart failure therapies in clinical practice: vital to modern medicine. Cardiology Plus. 2024;9(1):6–8. doi: 10.1097/CP9.0000000000000073.

[26] Vardeny O, Claggett B, Kachadourian J, et al. Incidence, predictors, and outcomes associated with hypotensive episodes among heart failure patients receiving sacubitril/valsartan or enalapril: the PARADIGM-HF trial (prospective comparison of angiotensin receptor neprilysin inhibitor with angiotensin-converting enzyme inhibitor to determine impact on global mortality and morbidity in heart failure). Circ Heart Fail. 2018;11(4):e004745. doi: 10.1161/CIRCHEARTFAILURE.117.004745. 29643067

[27] Mebazaa A, Davison B, Chioncel O, et al. Safety, tolerability and efficacy of up-titration of guideline-directed medical therapies for acute heart failure (STRONG-HF): a multinational, open-label, randomised, trial. Lancet. 2022;400(10367):1938–1952. doi: 10.1016/S0140-6736(22)02076-1. 36356631

[28] Divisón-Garrote JA, de la Cruz JJ, de la Sierra A, et al. Prevalence of office and ambulatory hypotension in treated hypertensive patients with coronary disease. Hypertens Res. 2020;43(7):696–704. doi: 10.1038/s41440-020-0462-9. 32398795

[29] Bangalore S, Qin J, Sloan S, et al. What is the optimal blood pressure in patients after acute coronary syndromes?: relationship of blood pressure and cardiovascular events in the PRavastatin OR atorVastatin Evaluation and Infection Therapy-Thrombolysis In Myocardial Infarction (PROVE IT-TIMI) 22 trial. Circulation. 2010;122(21):2142–2151. doi: 10.1161/CIRCULATIONAHA.109.905687. 21060068

[30] Vidal-Petiot E, Ford I, Greenlaw N, et al. Cardiovascular event rates and mortality according to achieved systolic and diastolic blood pressure in patients with stable coronary artery disease: an international cohort study. Lancet. 2016;388(10056):2142–2152. doi: 10.1016/S0140-6736(16)31326-5. 27590221

[31] Böhm M, Young R, Jhund PS, et al. Systolic blood pressure, cardiovascular outcomes and efficacy and safety of sacubitril/valsartan (LCZ696) in patients with chronic heart failure and reduced ejection fraction: results from PARADIGM-HF. Eur Heart J. 2017;38(15):1132–1143. doi: 10.1093/eurheartj/ehw570. 28158398 PMC6251522

[32] Serenelli M, Jackson A, Dewan P, et al. Mineralocorticoid receptor antagonists, blood pressure, and outcomes in heart failure with reduced ejection fraction. JACC Heart Fail. 2020;8(3):188–198. doi: 10.1016/j.jchf.2019.09.011. 31926854 PMC7086149

[33] Mancia G. White coat effect. Innocuous or adverse phenomenon? Eur Heart J. 2000;21(20):1647–1648. doi: 10.1053/euhj.2000.2337. 11032689

[34] Grassi G, Mancia G, Esler M. Central and peripheral sympathetic activation in heart failure. Cardiovasc Res. 2022;118(8):1857–1871. doi: 10.1093/cvr/cvab222. 34240147

[35] Lohse MJ, Engelhardt S, Eschenhagen T. What is the role of beta-adrenergic signaling in heart failure? Circ Res. 2003;93(10):896–906. doi: 10.1161/01.RES.0000102042.83024.CA. 14615493

[36] Nardone M, Notarius CF, Badrov MB, et al. Attenuated sympathetic blood pressure transduction in patients with treated heart failure with reduced ejection fraction. Hypertension. 2022;79(12):2764–2773. doi: 10.1161/HYPERTENSIONAHA.122.19850. 36252088

[37] Ricci F, De Caterina R, Fedorowski A. Orthostatic hypotension: epidemiology, prognosis, and treatment. J Am Coll Cardiol. 2015;66(7):848–860. doi: 10.1016/j.jacc.2015.06.1084. 26271068

[38] Kario K, Williams B. Nocturnal hypertension and heart failure: mechanisms, evidence, and new treatments. Hypertension. 2021;78(3):564–577. doi: 10.1161/HYPERTENSIONAHA.121.17440. 34225469

