# Supplementary material for: Identification and prognosis of low office and ambulatory blood pressure in patients with heart failure
Source: Ann Med. 2025 Nov 7;57(1):2583558. doi: 10.1080/07853890.2025.2583558 (PMC12599569; doi:10.1080/07853890.2025.2583558)
Supplement: Supplemental Material [file IANN_A_2583558_SM5373.zip › suppl/Supplemental Material_clean_2.docx]

**Supplemental material**

Table S1 Characteristics of the study population by office SBP

Table S2 Multivariate Cox regression for the association of the primary outcome with office and 24-hour SBP

Table S3 Univariate Cox regression for the association of the primary outcome with other risk factors

Table S4 Characteristics of the patients included in GDMTs titration analysis by SBP groups

Table S5 ACEI/ARB/ARNI dose trajectories by SBP groups

Table S6 Univariate logistic regression for failure of ACEI/ARB/ARNI target dose achievement with other risk factors

Table S7 The specific events for the outcomes by SBP groups

Table S8 The association of the failure of ACEI/ARB/ARNI target dose achievement and the primary outcome with SBP groups in HFrEF and HFpEF

Table S9 Cox regression for the primary outcome by SBP groups with 130 mmHg as low office SBP threshold

Captions/legends of the supplementary figures are retained within the manuscript.

**Table S1** Characteristics of the study population by office SBP

|  |  | **Office SBP (mmHg)** | |  |
| --- | --- | --- | --- | --- |
|  | **Total (N=491)** | **< 120 (n=170)** | **≥ 120 (n=321)** | ***P*** |
| **Age, years** | 68.8 ± 16.8 | 67.1 ± 17.0 | 69.7 ± 16.7 | 0.73 |
| **Sex, male (%)** | 280 (57.0) | 92 (54.1) | 188 (58.6) | 0.34 |
| **NYHA Class, III/IV (%)** | 315 (64.2) | 119 (70.8) | 196 (61.4) | 0.05 |
| **Smoking (%)** | 71 (14.5) | 25 (14.7) | 46 (14.3) | 0.91 |
| **Comorbidities (%)** |  |  |  |  |
| Ischemic heart disease | 216 (44.0) | 67 (39.4) | 149 (46.4) | 0.14 |
| Hypertension | 325 (66.2) | 87 (51.2) | 238 (74.1) | <0.001 |
| Diabetes | 150 (30.5) | 52 (30.6) | 98 (30.5) | 0.99 |
| Atrial fibrillation | 179 (36.5) | 79 (46.5) | 100 (31.2) | 0.001 |
| **Laboratory measurements** |  |  |  |  |
| Hemoglobin, g/L | 128.5 ± 23.0 | 127.8 ± 23.9 | 128.9 ± 22.6 | 0.61 |
| eGFR, ml/min/1.73m^2^ | 73.0 ± 23.1 | 74.7 ± 23.5 | 72.1 ± 22.9 | 0.25 |
| Serum sodium, mmol/L | 140.7 ± 3.5 | 140.8 ± 3.2 | 140.7 ± 3.6 | 0.72 |
| Serum potassium, mmol/L | 4.0 ± 0.4 | 3.9 ± 0.5 | 4.0 ± 0.4 | 0.72 |
| Fasting glucose, mmol/L | 6.0 ± 2.5 | 5.8 ± 2.0 | 6.1 ± 2.7 | 0.19 |
| Total cholesterol, mmol/L | 4.2 ± 1.3 | 4.0 ± 1.1 | 4.3 ± 1.4 | 0.06 |
| NT-proBNP, pg/mL | 1199.0 (513.0 - 2808.3) | 1629.0 (538.4 – 3472.5) | 1099.0 (496.2 – 2231.8) | 0.04 |
| **TTE parameters** |  |  |  |  |
| LVEDD, mm | 52.0 (46.0-61.0) | 52.0 (45.0-61.0) | 51.0 (47.0-61.0) | 0.64 |
| LVEF, % | 59.0 (41.0-65.0) | 59.0 (40.0-65.0) | 59.0 (42.0-65.0) | 0.74 |
| **BP parameters, mmHg** |  |  |  |  |
| Office SBP | 125.5 ± 19.9 | 105.4 ± 9.5 | 136.2 ± 15.2 | <0.001 |
| Office DBP | 71.2 ± 13.3 | 64.7 ± 10.3 | 74.6 ± 13.5 | <0.001 |
| 24-hour SBP | 118.2 ± 18.1 | 110.2 ± 17.0 | 122.5 ± 17.3 | <0.001 |
| 24-hour DBP | 66.3 ± 11.1 | 63.2 ± 9.6 | 67.9 ± 11.6 | <0.001 |
| Daytime SBP | 118.4 ± 18.7 | 110.4 ± 16.5 | 122.7 ± 18.4 | <0.001 |
| Daytime DBP | 66.9 ± 11.0 | 63.6 ± 9.8 | 68.7 ± 11.3 | <0.001 |
| Nighttime SBP | 117.7 ± 21.1 | 109.3 ± 20.3 | 122.1 ± 20.2 | <0.001 |
| Nighttime DBP | 65.6 ± 12.1 | 62.4 ± 11.0 | 67.2 ± 12.4 | <0.001 |
| **Medication at discharge (%)** |  |  |  |  |
| ACEI/ARB/ARNI | 427 (87.0) | 143 (84.1) | 284 (88.5) | 0.17 |
| ≥50% ACEI/ARB/ARNI target dose | 230 (46.8) | 58 (34.1) | 172 (53.6) | <0.001 |
| Beta-blockers | 405 (82.5) | 149 (87.6) | 256 (79.8) | 0.03 |
| MRA | 342 (69.7) | 126 (74.1) | 216 (67.3) | 0.12 |
| SGLT2i | 88 (17.9) | 34 (20.0) | 54 (16.8) | 0.38 |
| Diuretic | 308 (62.7) | 106 (62.4) | 202 (62.9) | 0.90 |
| Calcium channel blockers | 121 (24.6) | 20 (11.8) | 101 (31.5) | <0.001 |
| Nitrate | 102 (20.8) | 29 (17.1) | 73 (22.7) | 0.14 |

Values are expressed as mean ± SD, median (IQR) or n (%). Between-group differences were estimated by Students’ t-test, Mann–Whitney U test, or chi-square test as appropriate. Values of P < 0.05 were considered statistical significance.

Abbreviations: ACEI, angiotensin-converting enzyme inhibitors; ARB, angiotensin II receptor blockers; ARNI, angiotensin receptor–neprilysin inhibitors; DBP, diastolic blood pressure; eGFR, estimated glomerular filtration rate; LVEDD, left ventricular end-diastolic dimension; LVEF, left ventricular ejection fraction; MRA, mineralocorticoid receptor antagonists; NT-proBNP, N-terminal pro-brain natriuretic peptide; NYHA, New York Heart Association; SBP, systolic blood pressure; SGLT2i, sodium-glucose cotransporter-2 inhibitors; TTE, transthoracic echocardiography.

**Table S2** Cox regression for the association of the primary outcome with office and 24-hour SBP.

|  | **Unadjusted** | | | **Adjusted ^a^** | | |
| --- | --- | --- | --- | --- | --- | --- |
|  | **HR (95% CI)** | ***P*** | ***R^2^*** | **HR (95% CI)** | ***P*** | ***R^2^*** |
| **Office SBP, per 10 mmHg** | 0.94 (0.85-1.02) | 0.14 | 0.002 | 0.94 (0.85-1.03) | 0.20 | 0.088 |
| **24-hour SBP, per 10 mmHg** | 0.94 (0.85-1.03) | 0.16 | 0.002 | 0.88 (0.79-0.98) | 0.02 | 0.096 |

HRs for office and 24-hour SBP represent risk of every 10 mmHg increase for the corresponding variate. Values of P < 0.05 were considered to indicate statistical significance. R^2^ was obtained by Bootstrap validation with 200 resamples. ^a^ adjustment variables included age, NYHA class, hemoglobin, eGFR, NT-proBNP, and the use of beta-blockers, MRA, diuretics, and nitrate.

Abbreviations: CI, conference interval; HR, hazard ratio; SBP, systolic blood pressure.

**Table S3** Univariate Cox regression for the association of the primary outcome with other risk factors

|  | **HR (95% CI)** | ***P*** | ***R^2^*** |
| --- | --- | --- | --- |
| Age, per 1 year | 1.04 (1.02-1.05) | <0.001 | 0.060 |
| Sex | 1.11 (0.80-1.55) | 0.54 | 0.002 |
| NYHA Class | 2.74 (1.81-4.15) | <0.001 | 0.054 |
| Smoking | 0.90 (0.71-1.13) | 0.36 | 0.001 |
| Ischemic heart disease | 1.14 (0.82-1.58) | 0.45 | 0.001 |
| Hypertension | 1.15 (0.81-1.64) | 0.43 | 0.001 |
| Diabetes | 1.16 (0.81-1.66) | 0.41 | 0.001 |
| Atrial fibrillation | 1.25 (0.89-1.76) | 0.19 | 0.001 |
| Hemoglobin | 0.81 (0.68-0.96) | 0.01 | 0.013 |
| eGFR | 0.68 (0.57-0.82) | <0.001 | 0.030 |
| Serum sodium | 1.03 (0.85-1.25) | 0.76 | 0.002 |
| Serum potassium | 0.97 (0.82-1.15) | 0.73 | 0.001 |
| Fasting glucose | 1.04 (0.88-1.22) | 0.63 | 0.002 |
| Total cholesterol | 0.95 (0.79-1.14) | 0.58 | 0.003 |
| NT-proBNP ^a^ | 1.51 (1.25-1.83) | <0.001 | 0.030 |
| LVEDD ^a^ | 1.01 (0.84-1.22) | 0.91 | 0.002 |
| LVEF ^a^ | 1.00 (0.84-1.20) | 0.97 | 0.002 |
| ACEI/ARB/ARNI | 0.78 (0.50-1.23) | 0.29 | 0.001 |
| Beta-blockers | 0.65 (0.44-0.96) | 0.03 | 0.006 |
| MRA | 1.83 (1.23-2.71) | 0.003 | 0.015 |
| SGLT2i | 0.86 (0.54-1.37) | 0.53 | 0.001 |
| Diuretic | 2.09 (1.42-3.06) | <0.001 | 0.025 |
| Calcium channel blockers | 0.82 (0.54-1.25) | 0.35 | <0.001 |
| Nitrate | 1.67 (1.16-2.40) | 0.01 | 0.014 |

Values of P < 0.05 were considered to indicate statistical significance. R^2^ was obtained by Bootstrap validation with 200 resamples. ^a^ NT-proBNP, LVEDD, and LVEF were normalized by log_10_ transformation.

Abbreviations: ACEI, angiotensin-converting enzyme inhibitors; ARB, angiotensin II receptor blockers; ARNI, angiotensin receptor–neprilysin inhibitors; CI, conference interval; eGFR, estimated glomerular filtration rate; HR, hazard ratio; LVEDD, left ventricular end-diastolic dimension; LVEF, left ventricular ejection fraction; MRA, mineralocorticoid receptor antagonists; NT-proBNP, N-terminal pro-brain natriuretic peptide; NYHA, New York Heart Association; SBP, systolic blood pressure; SGLT2i, sodium-glucose cotransporter-2 inhibitors.

**Table S4** Characteristics of the patients included in GDMTs titration analysis by SBP groups

|  | **Sustained**  **low SBP**  **(n=90)** | **Masked**  **low SBP**  **(n=109)** | **No**  **low SBP**  **(n=153)** | ***P*** | ***P* ^a^** | ***P* ^b^** | ***P* ^c^** |
| --- | --- | --- | --- | --- | --- | --- | --- |
| **Age, years** | 65.2 ± 15.6 | 69.0 ± 16.9 | 71.2 ± 15.9 | 0.02 | 0.02 | 0.84 | 0.31 |
| **Sex, male (%)** | 57 (63.3) | 66 (60.6) | 80 (52.3) | 0.19 | NA | NA | NA |
| **NYHA Class, III/IV (%)** | 63 (70.0) | 66 (60.6) | 97 (63.4) | 0.37 | NA | NA | NA |
| **Smoking (%)** | 16 (17.8) | 11 (10.1) | 23 (15.0) | 0.28 | NA | NA | NA |
| **Comorbidities (%)** |  |  |  |  |  |  |  |
| Ischemic heart disease | 30 (33.3) | 48 (44.0) | 74 (48.4) | 0.07 | NA | NA | NA |
| Hypertension | 38 (42.2) | 68 (62.4) | 131 (85.6) | <0.001 | <0.001 | <0.001 | 0.02 |
| Diabetes | 24 (26.7) | 21 (19.3) | 65 (42.5) | <0.001 | 0.06 | <0.001 | 0.85 |
| Atrial fibrillation | 41 (45.6) | 39 (35.8) | 56 (36.6) | 0.29 | NA | NA | NA |
| **Laboratory measurements** |  |  |  |  |  |  |  |
| Hemoglobin, g/L | 130.6 ± 25.0 | 128.1 ± 24.3 | 128.2 ± 21.2 | 0.69 | 1.00 | 1.00 | 1.00 |
| eGFR, ml/min/1.73m^2^ | 74.8 ± 22.9 | 72.1 ± 22.0 | 72.8 ± 23.3 | 0.71 | 1.00 | 1.00 | 1.00 |
| Serum sodium, mmol/L | 140.7 ± 3.4 | 140.7 ± 3.0 | 140.8 ± 3.6 | 0.91 | 1.00 | 1.00 | 1.00 |
| Serum potassium, mmol/L | 4.0 ± 0.5 | 4.0 ± 0.4 | 3.9 ± 0.4 | 0.14 | 0.56 | 0.19 | 1.00 |
| Fasting glucose, mmol/L | 5.6 ± 2.0 | 5.9 ± 2.2 | 6.2 ± 3.2 | 0.24 | 0.28 | 1.00 | 1.00 |
| Total cholesterol, mmol/L | 4.0 ± 1.0 | 4.0 ± 1.3 | 4.3 ± 1.2 | 0.10 | 0.25 | 0.19 | 1.00 |
| NT-proBNP, pg/mL | 1586.0 (601.8-3378.0) | 1184.0 (610.5-3014.8) | 1181.0 (558.5-2567.5) | 0.69 | NA | NA | NA |
| **TTE parameters** |  |  |  |  |  |  |  |
| LVEDD, mm | 54.0 (47.0-65.0) | 51.0 (48.0-63.0) | 50.5 (46.0-56.0) | 0.03 | 0.04 | 0.15 | 1.00 |
| LVEF, % | 52.0 (34.0-62.0) | 55.0 (39.0-64.0) | 63.0 (46.0-66.0) | <0.001 | 0.001 | 0.01 | 1.00 |
| **BP parameters, mmHg** |  |  |  |  |  |  |  |
| Office SBP | 105.2 ± 8.5 | 131.5 ± 10.8 | 131.5 ± 18.9 | <0.001 | <0.001 | 1.00 | <0.001 |
| Office DBP | 64.6 ± 10.2 | 74.1 ± 10.9 | 72.8 ± 14.1 | <0.001 | <0.001 | 1.00 | <0.001 |
| 24-hour SBP | 101.3 ± 9.5 | 109.0 ± 7.9 | 135.4 ± 13.2 | <0.001 | <0.001 | <0.001 | <0.001 |
| 24-hour DBP | 60.6 ± 7.3 | 64.1 ± 8.1 | 71.9 ± 11.3 | <0.001 | <0.001 | <0.001 | 0.03 |
| Daytime SBP | 101.7 ± 9.5 | 109.8 ± 8.3 | 135.5 ± 13.5 | <0.001 | <0.001 | <0.001 | <0.001 |
| Daytime DBP | 61.1 ± 7.8 | 64.9 ± 8.9 | 72.3 ± 11.6 | <0.001 | <0.001 | <0.001 | 0.02 |
| Nighttime SBP | 99.8 ± 11.6 | 107.4 ± 11.3 | 136.1 ± 17.9 | <0.001 | <0.001 | <0.001 | 0.001 |
| Nighttime DBP | 59.3 ± 8.0 | 62.5 ± 8.5 | 71.7 ± 13.2 | <0.001 | <0.001 | <0.001 | 0.12 |
| **Medication at discharge, (%)** |  |  |  |  |  |  |  |
| ACEI/ARB/ARNI | 79 (87.8) | 99 (90.8) | 138 (90.2) | 0.76 | NA | NA | NA |
| ≥50% ACEI/ARB/ARNI target dose | 29 (32.2) | 50 (45.9) | 100 (65.4) | <0.001 | <0.001 | 0.008 | 0.21 |
| Beta-blockers | 81 (90.0) | 90 (82.6) | 122 (79.7) | 0.12 | NA | NA | NA |
| MRA | 63 (70.0) | 76 (69.7) | 107 (69.9) | 1.00 | NA | NA | NA |
| SGLT2i | 20 (22.2) | 15 (13.8) | 26 (17.0) | 0.29 | NA | NA | NA |
| Diuretic | 48 (53.3) | 72 (66.1) | 108 (70.6) | 0.02 | 0.03 | 1.00 | 0.28 |
| Calcium channel blockers | 8 (8.9) | 20 (18.3) | 70 (45.8) | <0.001 | <0.001 | <0.001 | 0.26 |
| Nitrate | 8 (8.9) | 17 (15.6) | 53 (34.6) | <0.001 | <0.001 | 0.003 | 0.68 |

Values are expressed as mean ± SD, median (IQR) or n (%). Between-group differences were estimated by one-way analysis of variance (ANOVA), Kruskal-Wallis test, or chi-square test with the Bonferroni method for post-hoc analysis. Values of P < 0.05 were considered statistical significance. ^a^ Sustained low SBP vs No low SBP. ^b^ Masked low SBP vs No low SBP. ^c^ Sustained low SBP vs Masked low SBP.

Abbreviations: ACEI, angiotensin-converting enzyme inhibitors; ARB, angiotensin II receptor blockers; ARNI, angiotensin receptor–neprilysin inhibitors; DBP, diastolic blood pressure; eGFR, estimated glomerular filtration rate; LVEDD, left ventricular end-diastolic dimension; LVEF, left ventricular ejection fraction; MRA, mineralocorticoid receptor antagonists; NT-proBNP, N-terminal pro-brain natriuretic peptide; NYHA, New York Heart Association; SBP, systolic blood pressure; SGLT2i, sodium-glucose cotransporter-2 inhibitors; TTE, transthoracic echocardiography.

**Table S5** ACEI/ARB/ARNI dose trajectories by SBP groups

| **Dose trajectory groups, No (%)** | **Sustained low SBP** | **Masked**  **low SBP** | **No**  **low SBP** | **P** | ***P* ^a^** | ***P* ^b^** | ***P* ^c^** |
| --- | --- | --- | --- | --- | --- | --- | --- |
| Discontinuation/decrease | 6 (6.7) | 12 (11.0) | 8 (5.2) | 0.20 | NA | NA | NA |
| Unchanged and suboptimal-target dose | 52 (57.8) | 58 (53.2) | 67 (43.8) | 0.08 | NA | NA | NA |
| Initiation/increase but suboptimal-target dose | 15 (16.7) | 17 (15.6) | 10 (6.5) | 0.02 | 0.07 | 0.09 | 1.00 |
| Target dose achievement | 17 (18.9) | 22 (20.2) | 68 (44.4) | <0.001 | <0.001 | <0.001 | 1.00 |

Values are expressed as n (%). Between-group differences were estimated by chi-square test with Bonferroni method for post hoc. Values of P < 0.05 were considered statistical significance. ^a^ Sustained low SBP vs No low SBP. ^b^ Masked low SBP vs No low SBP. ^c^ Sustained low SBP vs Masked low SBP.

Abbreviations: SBP, systolic blood pressure.

**Table S6** Univariate logistic regression for failure of ACEI/ARB/ARNI target dose achievement with other risk factors

|  | **OR (95% CI)** | ***P*** |
| --- | --- | --- |
| Age, per 1 year | 1.00 (0.99-1.01) | 0.84 |
| Sex | 1.07 (0.68-1.70) | 0.76 |
| NYHA Class | 1.10 (0.69-1.77) | 0.68 |
| Smoking | 0.74 (0.40-1.39) | 0.35 |
| Ischemic heart disease | 1.07 (0.68-1.69) | 0.78 |
| Hypertension | 0.36 (0.21-0.63) | <0.001 |
| Diabetes | 0.76 (0.47-1.22) | 0.26 |
| Atrial fibrillation | 1.21 (0.76-1.94) | 0.43 |
| Hemoglobin | 0.92 (0.73-1.17) | 0.50 |
| eGFR | 0.86 (0.68-1.08) | 0.20 |
| Serum sodium | 0.96 (0.76-1.21) | 0.74 |
| Serum potassium | 1.05 (0.84-1.32) | 0.67 |
| Fasting glucose | 1.00 (0.79-1.27) | 1.00 |
| Total cholesterol | 0.92 (0.73-1.16) | 0.48 |
| NT-proBNP **^a^** | 1.21 (0.96-1.53) | 0.10 |
| LVEDD **^a^** | 9.61 (0.78-118.23) | 0.08 |
| LVEF **^a^** | 0.75 (0.57-0.98) | 0.04 |
| Beta-blockers | 1.11 (0.61-2.02) | 0.74 |
| MRA | 1.19 (0.73-1.94) | 0.48 |
| SGLT2i | 1.16 (0.63-2.14) | 0.64 |
| Diuretic | 0.86 (0.53-1.38) | 0.51 |
| Calcium channel blockers | 0.38 (0.23-0.62) | <0.001 |
| Nitrate | 0.72 (0.43-1.23) | 0.23 |

Values of P < 0.05 were considered statistical significance. **^a^** NT-proBNP, LVEDD, and LVEF were normalized by log_10_ transformation.

Abbreviations: ACEI, angiotensin-converting enzyme inhibitors; ARB, angiotensin II receptor blockers; ARNI, angiotensin receptor–neprilysin inhibitors; eGFR, estimated glomerular filtration rate; LVEDD, left ventricular end-diastolic dimension; LVEF, left ventricular ejection fraction; MRA, mineralocorticoid receptor antagonists; NT-proBNP, N-terminal pro-brain natriuretic peptide; NYHA, New York Heart Association; SBP, systolic blood pressure; SGLT2i, sodium-glucose cotransporter-2 inhibitors.

Table S7 The specific events for the outcomes by SBP groups.

| **Events, No (%)** | **Sustained low SBP** | **Masked low SBP** | **No low SBP** |
| --- | --- | --- | --- |
| Cardiac death | 16 (12.9) | 12 (7.9) | 16 (7.4) |
| Non-cardiac death | 7 (5.6) | 7 (4.6) | 12 (5.6) |
| HF rehospitalization | 26 (21.0) | 33 (21.9) | 28 (13.0) |

Values are expressed as n (%). The percentages represent the proportion of events relative to the total number of patients in each SBP group.

Abbreviations: HF, heart failure; SBP, systolic blood pressure.

**Table S8** The association of the failure of ACEI/ARB/ARNI target dose achievement and the primary outcome with SBP groups in HFrEF and HFpEF

| **HFrEF (n=118)** | | | | | |
| --- | --- | --- | --- | --- | --- |
|  | No. (%) | Failure of ACEI/ARB/ARNI  target dose achievement ^a^ | | Primary outcome **^b^** | |
|  |  | OR (95% CI) | *P* | HR (95% CI) | *P* |
| **No low SBP** | 41 (32.5) | Reference | NA | Reference | NA |
| **Sustained low SBP** | 40 (31.7) | 7.30 (2.09–30.68) | <0.01 | 3.82 (1.64–8.88) | <0.01 |
| **Masked low SBP** | 37 (29.4) | 7.00 (2.16–27.62) | <0.01 | 2.71 (1.13–6.54) | 0.03 |
| **HFpEF (n=314)** | | | | | |
|  | No. (%) | Failure of ACEI/ARB/ARNI  target dose achievement ^a^ | | Primary outcome **^b^** | |
|  |  | OR (95% CI) | *P* | HR (95% CI) | *P* |
| **No low SBP** | 154 (47.7) | Reference | NA | Reference | NA |
| **Sustained low SBP** | 70 (21.5) | 2.64 (1.29–5.71) | 0.01 | 1.74 (1.03–2.94) | 0.04 |
| **Masked low SBP** | 97 (27.7) | 1.97 (1.09–5.71) | 0.03 | 1.49 (0.93–2.40) | 0.06 |

Logistic regression for the failure of ACEI/ARB/ARNI target dose achievement and Cox Regression for the Primary Outcome with SBP groups in HFrEF and HFpEF. Statistical significance was set at P < 0.05. ^a^ adjusted variables included hypertension, LVEF, and CCB use. **^b^** adjustment variables included age, NYHA class, hemoglobin, eGFR, NT-proBNP, and the use of beta-blockers, MRA, diuretics, and nitrate.

Abbreviations: CI, conference interval; HFpEF, heart failure with preserved ejection fraction; HFrEF, heart failure with reduced ejection fraction; HR, hazard ratio; OR, odds ratio; SBP, systolic blood pressure.

**Table S9** Cox regression for the primary outcome by SBP groups with 130 mmHg as low office SBP threshold

|  | **HR (95% CI)** | ***P*** |
| --- | --- | --- |
| **No low SBP** | Reference | NA |
| **Sustained low SBP** | 1.97 (1.30-2.99) | 0.001 |
| **Masked low SBP** | 2.01 (1.20-3.36) | 0.01 |

Multivariate Cox regression for the primary outcome was adjusted for age, NYHA class, hemoglobin, eGFR, NT-proBNP, and the use of beta-blocker, MRA, diuretic, and nitrate. Values of P < 0.05 were considered to indicate statistical significance.

Abbreviations: CI, conference interval; HR, hazard ratio.
