# Supplementary material for: Identification and prognosis of low office and ambulatory blood pressure in patients with heart failure
Source: Ann Med. 2025 Nov 7;57(1):2583558. doi: 10.1080/07853890.2025.2583558 (PMC12599569; doi:10.1080/07853890.2025.2583558)
Supplement: Supplemental Material [file IANN_A_2583558_SM5373.zip › suppl/suppp_Fig_caption.docx]

**Fig. S1** Flow diagram of the study

Abbreviations: ABPM, ambulatory blood pressure monitoring; GDMTs, guideline-directed medical therapies; HF, heart failure.

**Fig. S2** Restricted cubic spline curves illustrating shape of the association between office/24-hour DBP and the primary outcome

Restricted cubic spline curves depicting the nonlinear relationship (nonlinearity P=0.01) of office DBP (A) and linear relationship (nonlinearity P=0.64) of 24-hour DBP (B) with the primary outcome of all-cause mortality and HF rehospitalization, adjusted for age, NYHA class, hemoglobin, eGFR, NT-proBNP, and the use of beta-blocker, MRA, diuretic, and nitrate. Solid lines represent HRs, and shaded areas represent 95% CIs.

CI, confidence interval; DBP, diastolic blood pressure; HR, hazard ratio.

**Fig. S3** Bar graphs showing individual changes in office SBP by SBP groups after 3-month GDMTs titration

Differences were found in the office SBP change after 3-month GDMTs titration among patients with sustained, masked, or no low SBP.

SBP, systolic blood pressure.

**Fig. S4** Forest plot illustrating the subgroup analysis of all-cause mortality and HF rehospitalization across different patient characteristics

Subgroup analysis of the primary outcome, adjusted for age, NYHA class, hemoglobin, eGFR, NT-proBNP, and the use of beta-blocker, MRA, diuretic, and nitrate. P for interaction was calculated between SBP groups and subgroup variables: gender, NYHA class, hypertension, diabetes, atrial fibrillation, eGFR (<60 vs ≥60 mL/min/1.73m²), and LVEF (<45% vs ≥45%). No evidence of heterogeneity was showed in different subgroups. HRs were for sustained and masked low SBP group compared to the no low SBP group, respectively. Values P < 0.05 were considered to indicate statistical significance.

CI, confidence interval; eGFR, estimated glomerular filtration rate; HR, hazard ratio; LVEF, left ventricular ejection fraction; NYHA, New York Heart Association.
