# Supplementary figures and images for: Identification and prognosis of low office and ambulatory blood pressure in patients with heart failure
Source: Ann Med. 2025 Nov 7;57(1):2583558. doi: 10.1080/07853890.2025.2583558 (PMC12599569; doi:10.1080/07853890.2025.2583558)

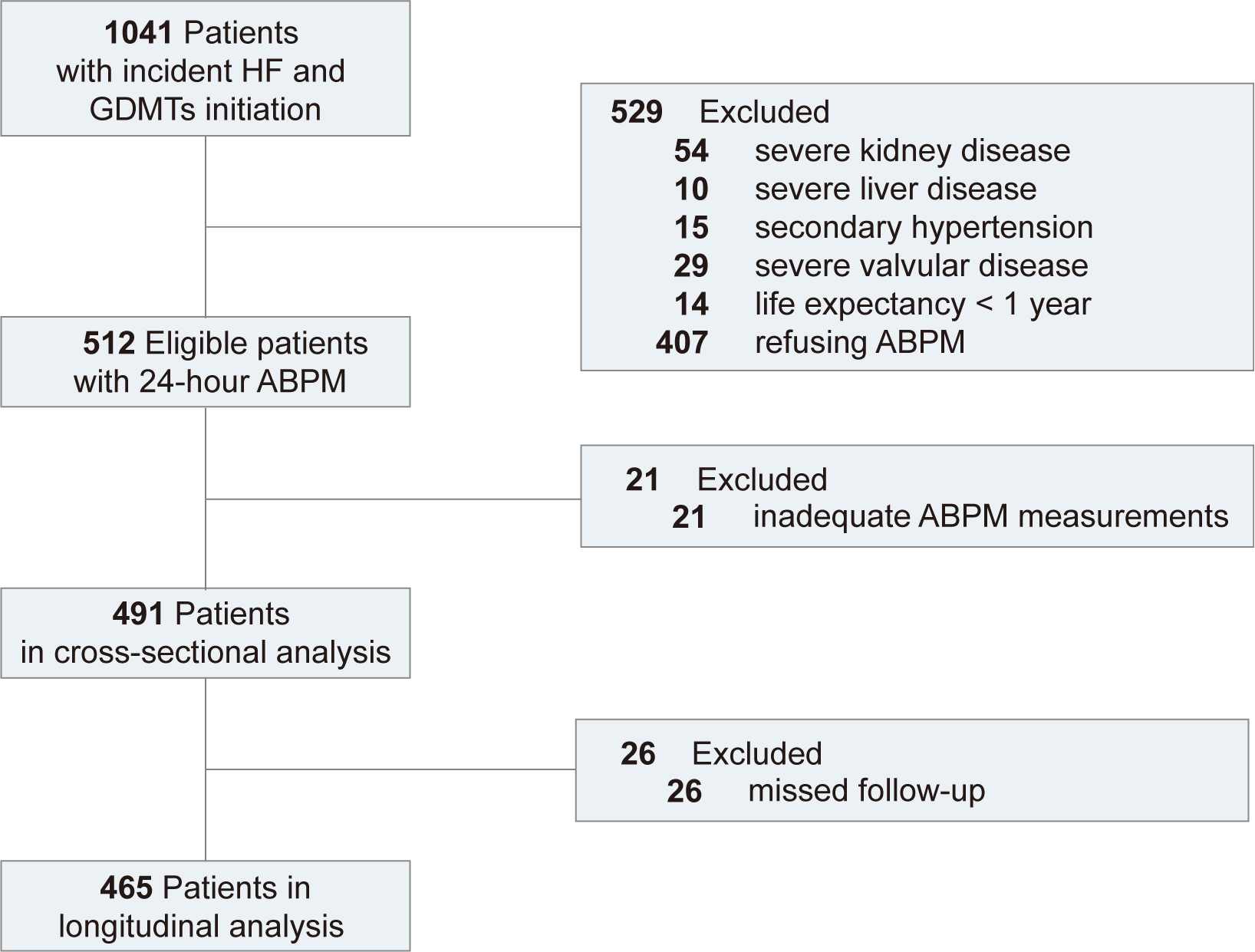

Supplement: Supplemental Material [file IANN_A_2583558_SM5373.zip › suppl/Figure_S1.tif]

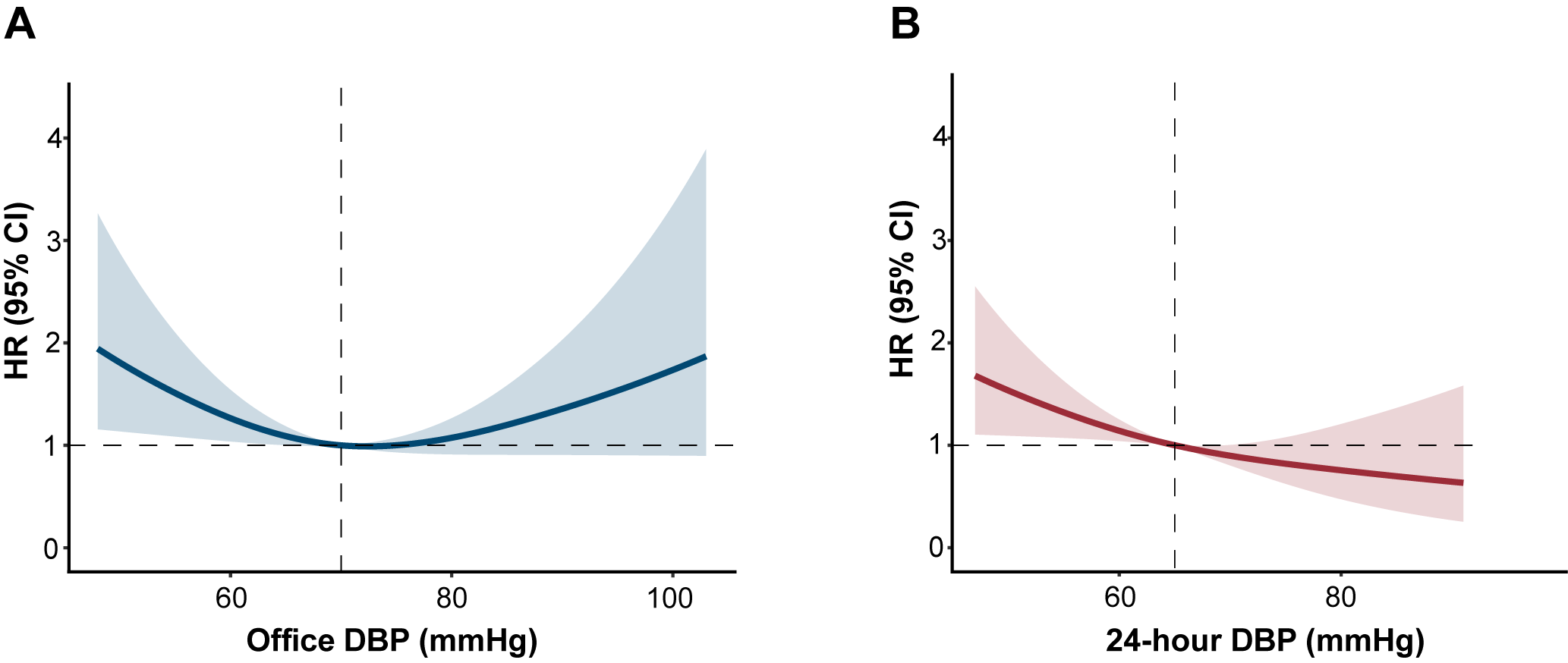

Supplement: Supplemental Material [file IANN_A_2583558_SM5373.zip › suppl/Figure_S2.tif]

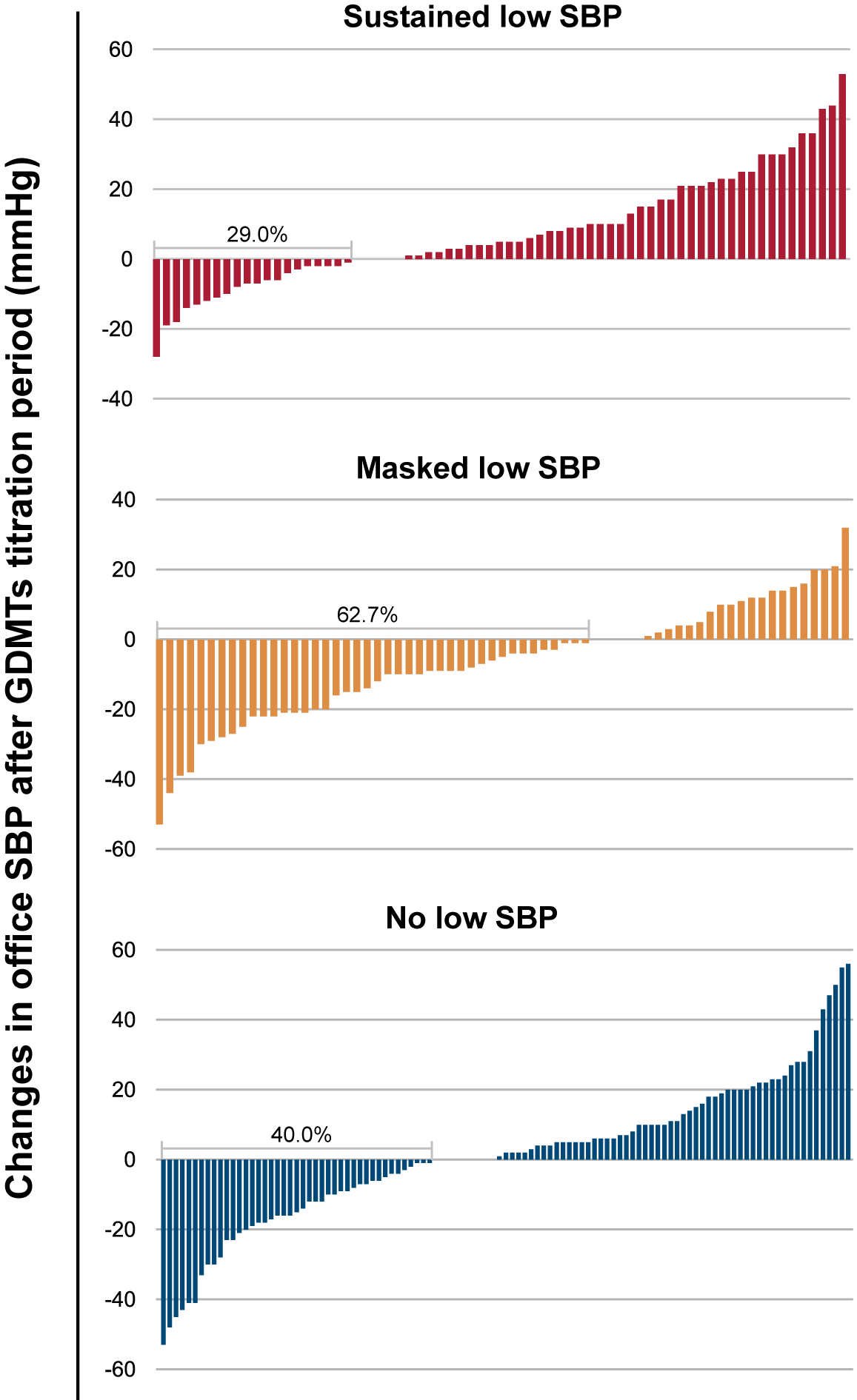

Supplement: Supplemental Material [file IANN_A_2583558_SM5373.zip › suppl/Figure_S3 (1).tif]

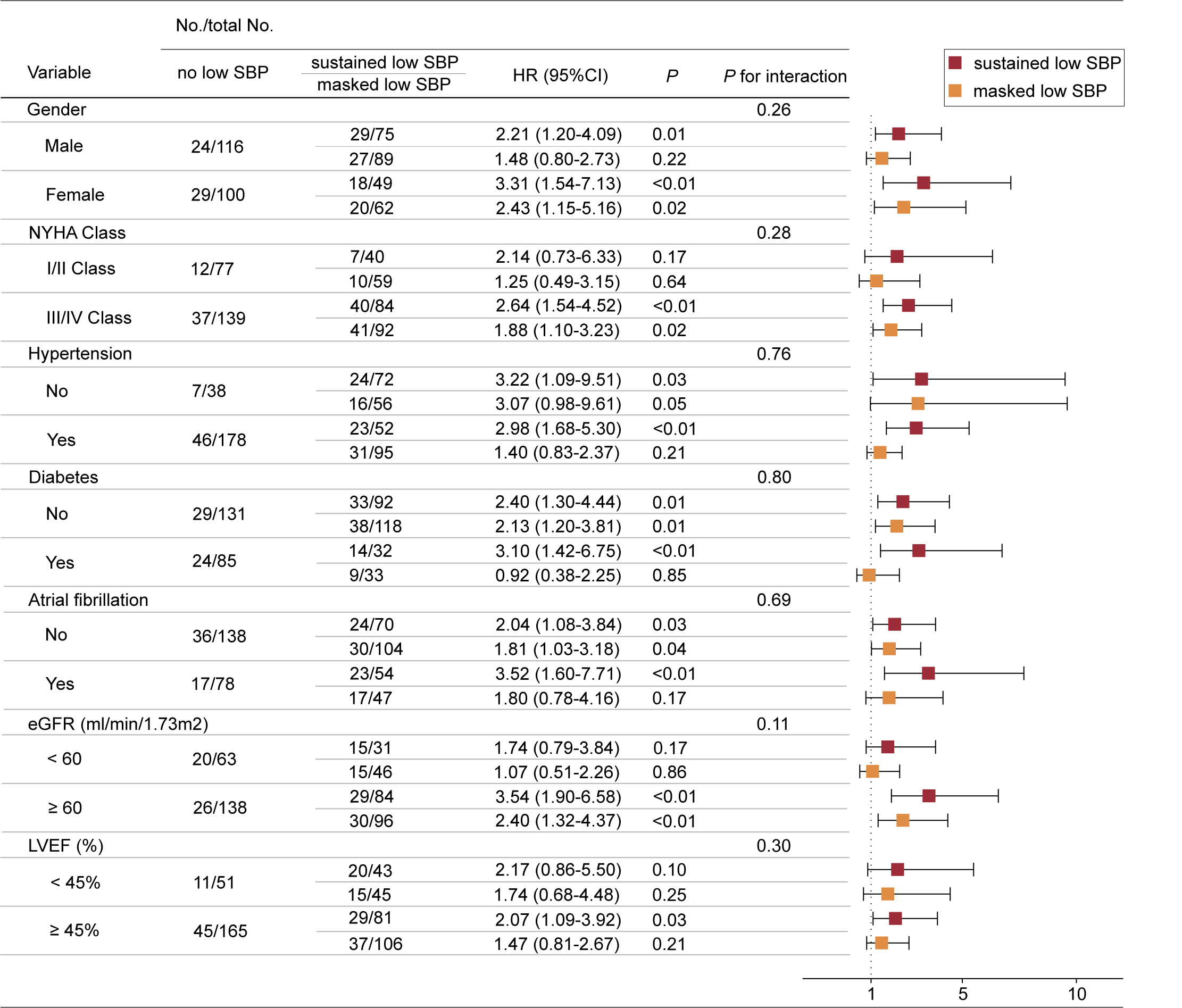

Supplement: Supplemental Material [file IANN_A_2583558_SM5373.zip › suppl/Figure_S4.tif]
